# SNAI1 expression and the mesenchymal phenotype: an immunohistochemical study performed on 46 cases of oral squamous cell carcinoma

**DOI:** 10.1186/1472-6890-10-1

**Published:** 2010-02-05

**Authors:** Joerg Schwock, Grace Bradley, James C Ho, Bayardo Perez-Ordonez, David W Hedley, Jonathan C Irish, William R Geddie

**Affiliations:** 1 Department of Laboratory Medicine & Pathobiology, Princess Margaret Hospital, University of Toronto, Toronto, Ontario, Canada; 2 Faculty of Dentistry, Princess Margaret Hospital, University of Toronto, Toronto, Ontario, Canada; 3 Division of Applied Molecular Oncology, Princess Margaret Hospital, University of Toronto, Toronto, Ontario, Canada; 4 Department of Otolaryngology, Head and Neck Surgery, Princess Margaret Hospital, University of Toronto, Toronto, Ontario, Canada

## Abstract

**Background:**

SNAI1 can initiate epithelial-mesenchymal transition (EMT), leading to loss of epithelial characteristics and, in cancer, to invasion and metastasis. We hypothesized that SNAI1 reactivation occurs in oral squamous cell carcinoma (OSCC) where it might also be associated with focal adhesion kinase (FAK) expression and p63 loss.

**Methods:**

Immunohistochemistry was performed on 46 tumors and 26 corresponding lymph node metastases. Full tissue sections were examined to account for rare and focal expression. Clinical outcome data were collected and analyzed.

**Results:**

SNAI1-positivity (nuclear, ≥ 5% tumor cells) was observed in 10 tumors and 5 metastases (n = 12 patients). Individual SNAI1(+) tumor cells were seen in primary tumors of 30 patients. High level SNAI1 expression (>10% tumor cells) was rare, but significantly associated with poor outcome. Two cases displayed a sarcomatoid component as part of the primary tumor with SNAI1(+)/FAK(+)/E-cadherin(-)/p63(-) phenotype, but disparate phenotypes in corresponding metastases. All cases had variable SNAI1(+) stroma. A mesenchymal-like immunoprofile in primary tumors characterized by E-cadherin loss (n = 29, 63%) or high cytoplasmic FAK expression (n = 10, 22%) was associated with N(+) status and tumor recurrence/new primary, respectively.

**Conclusions:**

SNAI1 is expressed, although at low levels, in a substantial proportion of OSCC. High levels of SNAI1 may herald a poor prognosis and circumscribed SNAI1 expression can indicate the presence of a sarcomatoid component. Absence of p63 in this context does not exclude squamous tumor origin. Additional EMT inducers may contribute to a mesenchymal-like phenotype and OSCC progression.

## Background

Epithelial-mesenchymal transition (EMT) is a highly conserved embryological process that permits epithelial cells to dissolve their cell-cell connections and to remodel their polarity in order to acquire mesenchymal properties with migratory capability [1,2]. EMT can be induced by a variety of molecules characterized by one common activity which is the down-regulation of E-cadherin by transcriptional repression. The aberrant activation of this process has long been suspected to contribute to tumor progression, i.e. invasion and metastasis, which is responsible for the majority of deaths related to malignant neoplasms [3]. Although there is continued skepticism regarding the clinical relevance of the phenomenon [4], evidence pointing towards the contribution of EMT in human tumors has been increasingly accumulated [5,6]. Sophisticated techniques using *in vitro *systems and animal models have allowed researchers to elucidate many of the molecular mechanisms governing the phenotypic characteristics of cells [7,8]. However, our knowledge regarding the specifics of EMT in human neoplasms thus far remains patchy at best, a situation owing partially to the complexity of EMT regulation and the constraints posed by the use of human tissue, but also to the multitude of different study designs and analytical tools.

A recent review by Becker et al. has summarized the current state of knowledge with regard to the E-cadherin repressor SNAI1, the first EMT inducer originally implicated with tumor progression [9]. SNAI1 (snail homolog 1; also Snail, SNA, SNAH, SLUGH2, dJ710H13.1) belongs to a family of zinc-finger transcription factors which also comprises SNAI2 (Slug) and SNAI3. The transcriptional repressor SNAI1 is an essential molecule which contributes to a series of coordinated morphogenetic events that lead to mesoderm formation in metazoans [10]. Its expression can be activated by different signaling cascades including receptor tyrosine kinase (RTK) signaling which is commonly deregulated in cancer [10]. In addition SNAI1 initiates a transcriptional program that modulates other genes involved in cell differentiation, leading to a general downregulation of epithelial, and up-regulation of mesenchymal, characteristics. Binding of SNAI1 to E-box elements in the E-cadherin promoter region leads to transcriptional repression of the CDH1 gene and resulting loss of E-cadherin expression, which is considered a "hallmark" of EMT. Two other molecules previously linked to the loss of epithelial features and the acquisition of a mesenchymal-like phenotype in cancer cells are p63 and focal adhesion kinase (FAK). Downregulation of ΔNp63α, the predominant p63 isoform in squamous cell carcinoma, was recently shown to occur in response to SNAI1 and to increase SCC invasiveness [11-13]. No data examining this relationship in clinical tissues have been presented thus far even though p63 expression is routinely considered evidence of squamous differentiation in poorly differentiated carcinomas. FAK, in contrast, has previously been linked to epithelial-mesenchymal transition [14,15] and a migratory cellular phenotype in more general terms [16]. The kinase is an essential part of a signaling complex formed by Src and FAK which localizes to contact points between the cell and the extra-cellular matrix, called focal adhesions. Within these focal adhesions FAK contributes to a bi-directional signaling by its ability to integrate stimuli derived from the extracellular space *via *integrin receptors and RTKs as well as the intracellular space due to connections with the cytoskeleton. Signaling downstream of FAK involves a host of different molecules and pathways. The more prominent downstream mediators are Rho-family GTPases which impact on the composition of the cytoskeleton as well as the MAPK and the Akt/PKB pathway which regulate complex cellular functions such as proliferation and survival [16].

In this study we examined the expression of SNAI1, E-cadherin, FAK and p63 in a cohort of patients with squamous cell carcinoma of the oral cavity (OSCC) treated at a center of tertiary/quaternary care. Taking into account the focal nature of tumor-associated EMT we used full sections of both primary tumors and metastases to explore SNAI1-associated EMT by immunohistochemistry. We also examined the specificity of different commercially available SNAI1 antibodies, including one antibody which, to the best of our knowledge, has not been used previously for the study of SNAI1 expression in formalin-fixed paraffin-embedded (FFPE) clinical material.

## Methods

### Patients, Tissue Selection and Characteristics of Study Cohort

This retrospective study was approved by the institutional research ethics board of the University Health Network, Toronto, ON (REB protocol #06-0805-T) prior to initiation of any research activities. All requirements with regard to retrospective data collection as well as retrieval and use of archived tissue specimens were followed. Formalin-fixed paraffin-embedded (FFPE) tissue of a longitudinal convenience cohort of OSCC was retrieved from the UHN archive. 46/50 patients of the initial cohort had sufficient FFPE tissue available to be included in the study. All patients were diagnosed and received treatment at our institution between the years 1996 and 2000. For each patient one representative paraffin block from primary tumor and one lymph node metastasis were selected after review of H&E stained slides. Clinical information was accumulated from electronic as well as paper files and data collected by Cancer Care Ontario (Toronto, ON). 2/46 cases had pre-operative radiation therapy. The metastasis of one of these two cases was excluded from further analysis due to lack of viable tumor tissue leaving 25/26 metastases for immunohistochemical examination. One patient had a reported one-time history of OSCC 25 years prior to inclusion in this study. In 37/46 patients a current or former smoking history was reported. Other characteristics of the cohort are summarized in Table 1.

**Table 1:** Characteristics of the Study Cohort

Parameter	Measurement	Number of Cases (%)
**Patients**	**Count**	**46 **(100%)

**Age**	**Mean**	**60.2**
	**Median**	**63**
	**Range**	**34-82**

**Sex**	**Male**	**30**
	**Female**	**16**

**Follow Up (months)**	**Mean**	**44.4**
	**Median**	**21.5**
	**Range**	**2-141**

**Time to Event (months)**	**Mean**	**13**
	**Median**	**9**
	**Range**	**2-51**

**TNM**	**T1**	**6 **(13%)
	**T2**	**18 **(39%)
	**T3**	**9 **(20%)
	**T4**	**13 **(28%)
	**N0**	**20 **(44%)
	**N1**	**7 **(15%)
	**N2**	**19 **(41%)
	**N2a**	**2 **(4%)
	**N2b**	**14 **(30%)
	**N2c**	**3 **(7%)
	**M0**	**46 **(100%)

**Grade**	**G1**	**4 **(9%)
	**G2**	**37 **(80%)
	**G3**	**5 **(11%)

**Location**	**Tongue**	**27 **(59%)
	**Floor**	**5 **(11%)
	**Tongue+Floor**	**8 **(17%)
	**Right retromolar trigone**	**2 **(4%)
	**Left retromolar trigone**	**1 **(2%)
	**Right buccal**	**2 **(4%)
	**Left inferior alveolar**	**1 **(2%)

Adverse Event*	**Yes**	**25 **(54%) [2^nd ^primary: 4/25]
	**No**	**21 **(46%)

*Adverse Event: Tumor recurrence or second primary OSCC

### Immunohistochemistry (IHC)

Several commercially available anti-SNAI1 antibodies were evaluated for their applicability to paraffin-IHC (P-IHC). Further specificity testing then focused on SC10432 (goat polyclonal, [E-18]; St. Cruz Biotechnology, Santa Cruz, CA) and AF3639 (goat polyclonal; R&D Systems, Minneapolis, MN). Xenografts of two previously studied cell lines SiHa and ME180 obtained from the American Type Culture Collection (ATCC, Manassas, VA) were employed as controls for all staining procedures [17]. Although AE1/AE3-positive, both cell lines represent opposite ends of the epithelial-mesenchymal spectrum with an E-cadherin(-), p63(-), SNAI1(+), FAK(+) phenotype in SiHa and an E-cadherin(+), p63(+), SNAI1(-), FAK(+/-) phenotype in ME180 (Additional File 1: A). In addition, three different FFPE tissue samples were used for initial antibody testing: (i) 1^st ^trimester human placenta, (ii) SiHa xenograft tissue derived from SCID mice after s.c. suspension injection and (iii) tissue from a spindle cell carcinoma specimen of the piriform sinus with vimentin positivity and foci of osteosarcoma. To validate the nuclear SNAI1 staining seen with those tissues two tests were performed. First, SC10432 was tested in all specimens after prior incubation with the corresponding blocking peptide SC10432P at 1:10 protein ratio *versus *PBS (Additional File 1: B). Second, immunoblotting was performed on SiHa cell lysates treated with SNAI1 siRNA *versus *control. Immunoblots revealed a singular band at ~30 kDa with a clear decrease after treatment (Additional File 1: C). By IHC, an essentially identical nuclear reactivity was found for both SC10432 and AF3639. However, AF3639 in our hands produced less cytoplasmic staining and a stronger nuclear signal than SC10432 (Additional File 1: D).

Clinical FFPE material was cut at 4 μm thickness and processed with standard procedures. Microwave antigen retrieval was performed in 10 mM Citrate buffer, pH 6.0, for 10 min. The following antibodies and conditions were applied: SC10432 (1:200, overnight) and AF3639 (1:1000, overnight), rabbit polyclonal FAK (#3285, 1:200, overnight; Cell Signaling Technology, Danvers, MA), mouse monoclonal HMWK 34βE12 (M0630, 1:100, 1 hr; Dako, Mississauga, ON), mouse monoclonal Vimentin (Vim3B4, 1:200, 1 hr; American Research Products, Belmont, MA), mouse monoclonal E-cadherin (36B5, 1:100, overnight; Vector Labs, Burlington, ON), mouse monoclonal p63(7JUL, 1:50, 2 hrs; Vector Labs) and human cytokeratin cocktail (AE1/AE3, 1:200, 1 hr; Dako). Secondary antibodies were biotin-labeled IgG (Vector Labs). Linking reagents were Streptavidin-HRP (ID Labs, London, ON) and/or Streptavidin-AP (Dako). Labeling reagents were NovaRed (SK-4800, Vector Labs), DAB+AP double-labeling: DAB (K3466, Dako) & AP VectorRed (SK-5100, Vector Labs). Sections were counterstained with Gill modified hematoxylin and coverslipped with Permount* Mounting Medium (Fisher Scientific, Nepean, ON). First-trimester placenta and xenograft tissue were carried with every staining series as controls.

### Cell Culture, Transfection and Immunoblotting

SiHa and ME180 cells were cultured under recommended conditions. Xenografts were grown subcutaneously in SCID mice after suspension injection. Experimentation involving animals was done under protocols approved according to the regulations of the Canadian Council on Animal Care. Transfection of SiHa cells with On-Target*plus *SMARTpool human-SNAI1 siRNA (Dharmacon, Lafayette, CO) and On-Target*plus *siControl non-targeting siRNA was done using Oligofectamine™ reagent (Invitrogen, Burlington, ON) according to protocols supplied by the manufacturer. Western immunoblotting with anti-SNAI1 (AF3639, 1:1000, overnight, 4°C) was done as previously described [18].

### Scoring and Statistical Methods

IHC scoring was done by a pathologist-in-training (JS) and a board-certified pathologist (WRG). Scoring was performed on full sections to account for rare and localized events such as EMT at the tumor invasion front. A histology scoring system (H-score) was used to account for area and staining intensity since considerable heterogeneity was observed in individual sections:

E-cadherin, FAK, p63: 

Intensity Category 0

Intensity Category 1 × Area of Category 1 (%) +

Intensity Category 2 × Area of Category 2 (%) +

Intensity Category 3 × Area of Category 3 (%) = H-Score

FAK scoring was done for the predominant cytoplasmic compartment; however occasional nuclear staining was also seen. E-cadherin was scored with intensity 2 and 3 for weak and strong membrane staining, respectively. Cytoplasmic staining of any intensity without membrane labeling was considered intensity 1 [17]. P63 was scored for nuclear staining only, and no significant labeling of any other cell compartments was seen. SNAI1 staining was categorized as follows:

SNAI1: 

Category 0: no tumor cells with nuclear staining

Category 1: rare tumor cells with nuclear staining, less than 5%

Category 2: ≥ 5% tumor cells with nuclear staining (= SNAI1-positive)

SNAI1 in tumor-surrounding stroma was assessed using the following categories: absent (no SNAI1 even at 400×), rare/occasional cells (single cells can be found at 400×), frequent (SNAI1 positive stroma cells are readily detectable), abundant (positivity of a majority of stroma cells easily discernable at 100×).

Statistical analysis of categorical data was performed using Fisher's exact test. Spearman nonparametric correlation was used to assess the relationship between variables. Kaplan-Meier survival data were compared using the log-rank test. Two-tailed p values < 0.05 were considered statistically significant. Statistical software was GraphPad Prism, Version 5.01 (GraphPad Software, La Jolla, CA).

## Results

### SNAI1 Expression in Tumor Cells and Tumor-Associated Stroma of OSCC

**Figure 1:**
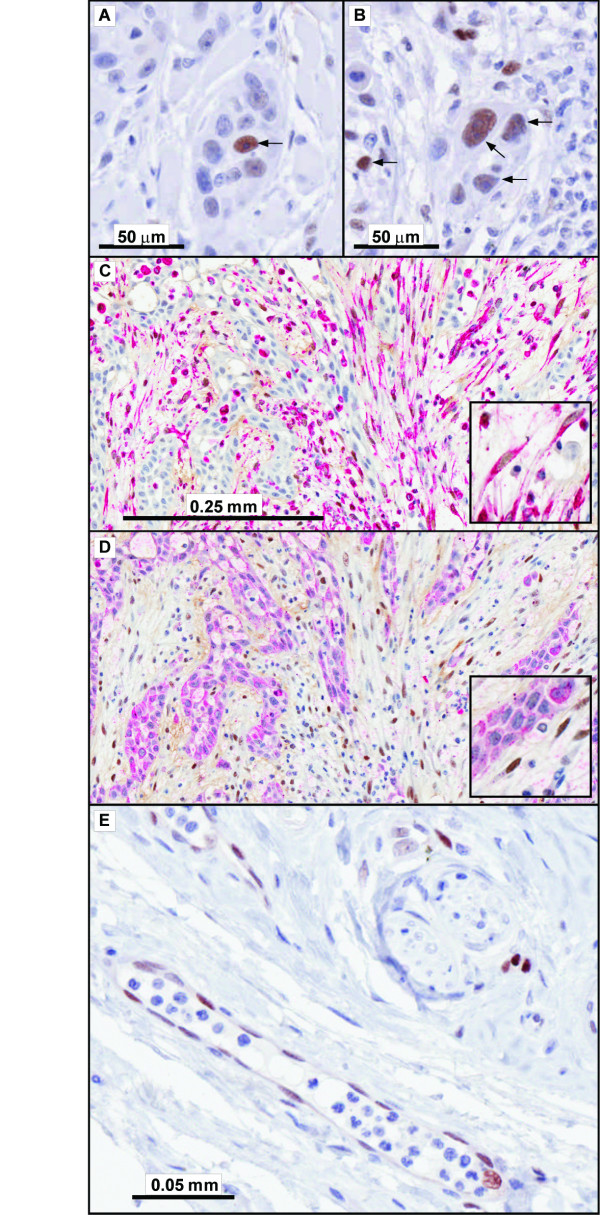
**SNAI1 expression occurs in tumor cells, tumor-associated stroma and endothelium**. **A**, SNAI1 expression (arrow) in an OSCC with 5% positivity in the tumor cells of the primary site. **B**, SNAI1 expression (arrows) in an OSCC with 20% positivity in the tumor cells of the primary site. **C**, Vimentin (cytoplasmic, red) and SNAI1 (nuclear, brown) show co-expression in stromal cells with fibroblastic morphology. **D**, HMWK (cytoplasmic, red) in strands of diffusely infiltrating OSCC and SNAI1 are mutually exclusive. **E**, SNAI1 in endothelium in the vicinity of the same OSCC shown in C and D.

Immunohistochemistry (IHC) for SNAI1 in clinical material was performed in two parallel series using both antibodies, SC10432 and AF3639. In specimens displaying SNAI1 nuclear positivity the same cell population was labeled by both antibodies. However, AF3639 produced strong nuclear staining with better signal-background-ratio. Thus, final scoring and assessment was performed based on AF3639 stained slides. SNAI1 expression in our series of OSCC was found to occur as an infrequent event that was mostly observed in few, scattered tumor cells representing less than 5% of the entire tumor population. These rare SNAI1(+) cells were frequently seen either in the vicinity of inflammation or close to the invasion front of the tumor. However, at this level SNAI1 expression was observed in the majority (30/46; 65%) cases. 10 (22%) cases displayed SNAI1 expression at a level of ≥ 5% (Category 2: SNAI1-positive) and 5 of these cases displayed ≥ 10% SNAI1 positivity in the primary tumor. 6 cases (13%) were entirely negative for SNAI1 in tumor cells. If lymph node metastases were considered in addition to the primary tumors, 2 more cases were determined to be SNAI1-positive resulting in a total of 12/46 (26%) cases. Overall 5/25 (20%) lymph node metastases were SNAI1-positive, 20/25 (80%) had less than 5% SNAI1 expression and none of the metastases was entirely negative for SNAI1. Of the SNAI1-positive metastases 3/5 were associated with SNAI1 expression in primary tumors and 2/5 cases were found SNAI1-positive in the metastasis only. Of the 10 cases categorized as SNAI1-positive based on the primary tumor, 6 had lymph node metastases at surgery. 5/6 of these lymph nodes were examined by IHC after exclusion of one tissue sample that lacked viable tumor. 3 of the remaining 5 metastases were categorized as SNAI1-positive, 2/5 contained rare (<5%) tumor cells expressing SNAI1. Two representative examples of SNAI1 expression in tumor cells are shown in Figure 1A and 1B.

SNAI1 nuclear staining was more frequent in stroma than in tumor cells. 6 (13%) cases were categorized as "abundant", 25 (54%) as "frequent" and 15 (33%) as "rare/occasional" for stromal SNAI1expression. All 6 cases of the stromal category "SNAI1-abundant" had SNAI1 expression at a <5% level in the corresponding tumor area. Of the 25 cases classified as "SNAI1-frequent" based on their stroma, 4/25 corresponding tumors were negative, 18/25 had SNAI1 expression in <5% tumors cells and 3/25 were categorized as SNAI1-positive (≥ 5%). Finally, in the group of cases with "rare/occasional" SNAI1 expression in tumor stroma, 2/15 corresponding tumors were negative, 6/15 had SNAI1 expression in <5% tumors cells and 7/15 were categorized as SNAI1-positive (≥ 5%). Chromatic double-staining for HMWK or Vimentin together with SNAI1 in several cases with prominent stromal expression indicated a mutually exclusive labeling between SNAI1 and HMWK, but coincidence of SNAI1 with Vimentin (Figure 1C and 1D). Among normal tissue constituents nuclear SNAI1 expression was most frequently seen in endothelial cells (Figure 1E), and there was an impression of a more frequent SNAI1 positivity in vascular elements closer to the tumor. The endothelial expression of SNAI1 was not included into the assessment of the stroma.

### Relationship between SNAI1 Expression and Clinical Parameters

**Figure 2:**
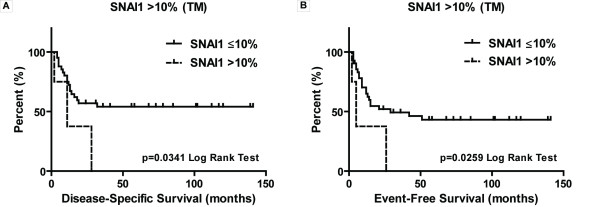
**High level SNAI1 expression, although infrequent, is associated with poorer outcome in OSCC**. **A**, Disease-specific survival and **B**, Event-free survival are significantly shorter (p = 0.0341 and p = 0.0259, respectively) in a small group of patients (n = 4) with high SNAI1 expression in tumor cells (>10%).

To examine the clinical impact of SNAI1 expression, the distribution of cases with Category 2 labeling and higher (≥ 5% or ≥ 10% SNAI1-positive) among different clinicopathological parameters was examined. No statistically significant relationships were found with patient gender, TNM stage, nodal status, grade, perineural and lymphovascular invasion as well as adverse events defined as recurrence or second primary OSCC. Next, we studied SNAI1 expression in tumor cells with regard to patient prognosis. No significant difference with regard to event-free survival (EFS; p = 0.3090) and disease-specific survival (DSS; p = 0.1613) was found for the cut-off used to define SNAI1-positive staining (Category 2: ≥ 5% tumor cells). However, a small group of cases with SNAI1-positivity in more than 10% tumor cells (n = 4/46) was characterized by both significantly shorter EFS and DSS (p = 0.0259 and p = 0.0341, respectively). Inclusion of 2 additional cases displaying SNAI1-positivity >10% in lymph node metastases defined a poor-prognosis group with significantly shorter EFS (median 5 *vs*. 42 months, p = 0.0007) and DSS (median 9 *vs*. undefined, p = 0.0005) (Figure 2 and Additional File 2).

Next, SNAI1 expression in tumor-associated stroma was examined with regard to outcome parameters. For the three different categories with "abundant", "frequent" and "rare/occasional" SNAI1 expression, 4/6 (67%), 14/25 (56%) and 7/15 (47%) adverse events as well as 4/6 (67%), 10/25 (40%) and 7/15 (47%) disease-specific deaths were present, respectively. Analysis of EFS and DSS did not reveal significant differences between these groups.

### Gain of FAK, Loss of E-cadherin and p63 Are Early Alterations in OSCC

Cytoplasmic FAK expression (FAK_c_) in cancer and metastases was mostly weak to moderate, and considerable intra-tumoral heterogeneity was observed. A similar situation was found for E-cadherin with areas of distinct membrane staining and areas of E-cadherin loss occasionally present in the same tumor. Overall, an inverse relationship was noted between FAK and E-cadherin expression (Additional File 3: A). This inverse relationship was statistically significant if all tissues including tumor-adjacent normal mucosa, primary tumors and metastases were considered (Spearman r:-0.6472, p < 0.0001), but not if the relationship was assessed for primary tumors alone (Spearman r: -0.1864, p = 0.2149). Comparing the H-scores for FAK and E-cadherin between the three different tissue compartments, a significant difference was noted for mucosa *versus *tumor or metastasis (Additional File 3: B+C). However, no significant difference was found between tumor and metastasis indicating that FAK gain and E-cadherin loss might be early events during tumor development. P63 was examined in the same manner and a more subtle, but significant loss of expression was found comparing the basal/parabasal compartment of normal mucosa with tumor or metastasis. Again there was no significant difference between tumor and metastases (Additional File 3: D).

**Figure 3:**
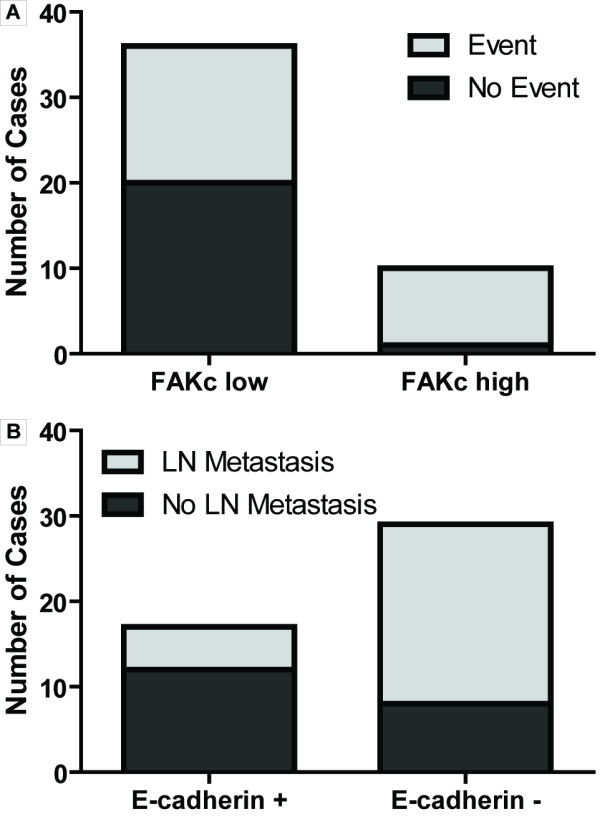
**FAK expression and E-cadherin loss are associated with adverse events and lymph node metastasis, respectively**. **A**, FAK_c _high (score ≥ 2 in >10% tumor area) cases have a significantly higher proportion of adverse events (tumor recurrence/2^nd ^primary tumor: 9/10) than FAK_c _low cases (16/36, p = 0.0105). **B**, Membranous E-cadherin(+) cases (score 3 in >10% tumor area) have a significantly lower proportion of lymph node metastases (5/17) than E-cadherin(-) cases (21/29, p = 0.0064).

Comparison between cases with strong cytoplasmic FAK staining (score ≥ 2 in >10% tumor area: FAK_c _high) and weak cytoplasmic FAK staining (score ≤ 1 in ≥ 90% tumor area: FAK_c _low) revealed a significantly higher proportion of adverse events (9/10, 90% *vs*. 16/36, 44%; p = 0.0105, Figure 3A), shorter event-free survival (EFS; FAK_c _high: median 9 months, n = 10 *vs*. FAK_c _low: median: undefined, n = 36; p = 0.0021) and a trend towards shorter overall survival (OS; FAK_c _high: median 11 months *vs*. FAK_c _low: median: 68 months; p = 0.0535) in the former group, indicating a link between the kinase and tumor progression. Furthermore, E-cadherin loss (Score ≤ 2 in ≥ 90% tumor area) was associated with a significantly higher proportion of positive nodal status at surgery (21/29, 72% *vs*. 5/17, 29%; p = 0.0064, Figure 3B). E-cadherin loss was not associated with shorter EFS and OS in our cohort. About twice the number of patients that suffered an adverse event had a history of positive nodal status at surgery (16/25, 64%) compared to only 9/25 (36%) patients who developed an event without prior lymph node metastasis. However, this relationship was not statistically significant (p = 0.3721).

### Two Cases with Sarcomatoid Component Suggest EMT in OSCC

**Figure 4:**
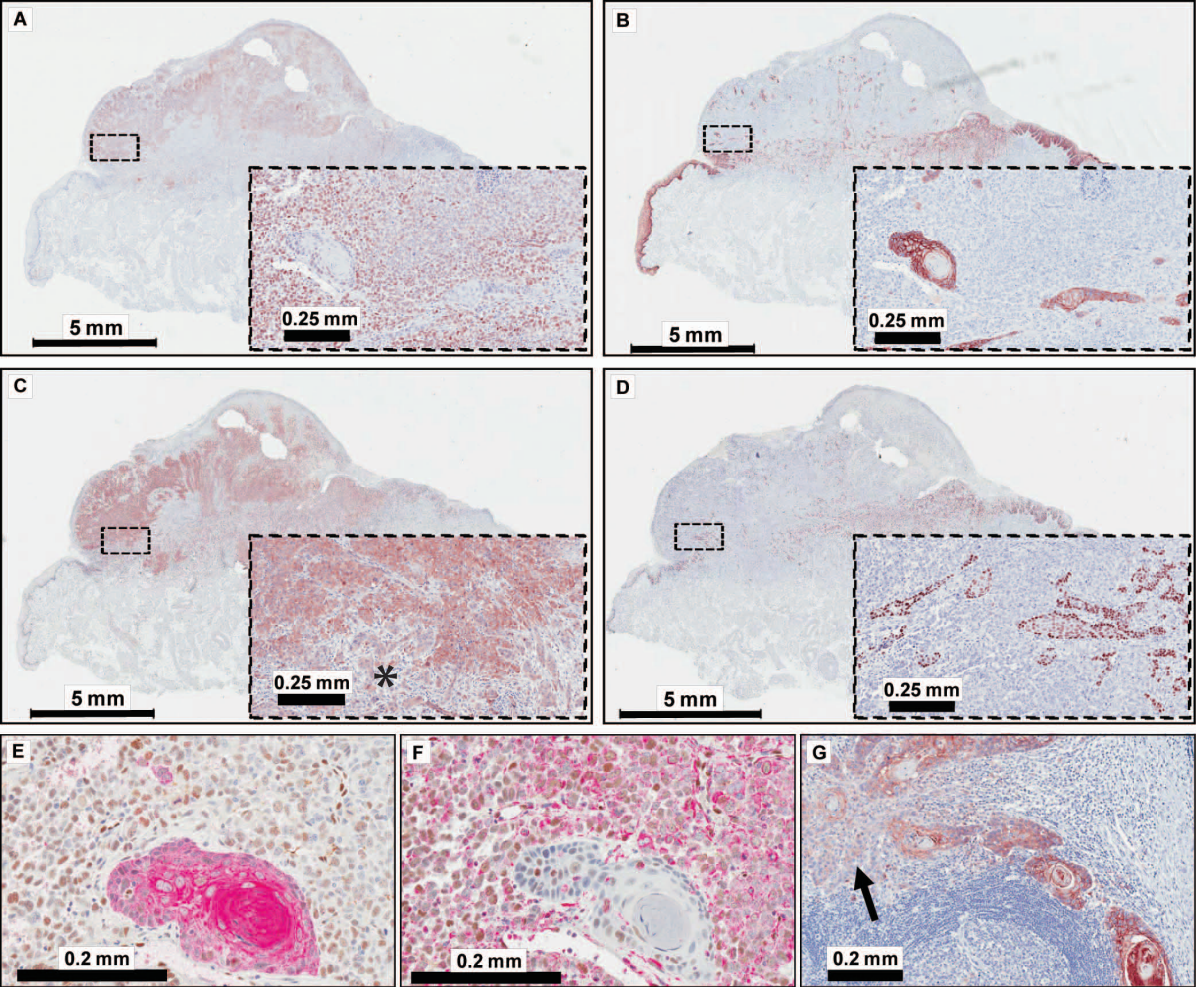
**Immunohistochemical profile of OSCC with sarcomatoid component, Case 1**. **A**, SNAI1; **B**, E-cadherin; **C**, FAK and **D**, p63 are differentially expressed in sarcomatoid (A+C) and squamous (B+D) component. Magnified inserts correspond to framed areas in the section overview. An asterisk in insert C indicates strands of squamous cell carcinoma intermingled with the sarcomatoid component. Dual staining of SNAI1 (nuclear, brown) and **E**, HMWK or **F**, Vimentin (red) illustrates the mesenchymal character of the sarcomatoid tumor component. **G**, Lymph node metastasis with focal E-cadherin loss (arrow).

Closer examination of the SNAI1(+) group of cases revealed 2 patients with distinct SNAI1-expressing tumor areas of 80% and 30% associated with p63 loss (Additional File 3: E). The first case was a 78 year old female patient with a T3N1M0 SCC of the tongue initially graded as G2-3. This patient suffered a local recurrence 2 months after surgery despite negative margins and lack of perineural as well as lymphatic/vascular invasion and died after a total follow-up of 4 months. The primary tumor in this case presented as exophytic mass with a minor component of distinctly squamous cell nests and a major, poorly differentiated component with E-cadherin(-), p63(-), SNAI1(+), FAK(+) phenotype (Figure 4 and Additional File 4). The nests of the squamous component displayed focal continuity from adjacent dysplastic epithelium and were found embedded into the sarcomatoid component throughout the tumor mass. Double labeling for SNAI1 with HMWK and SNAI1 with Vimentin confirmed the EMT phenotype of the sarcomatoid component with mutually exclusive staining for SNAI1 and HMWK. Weak AE1/AE3 staining was retained in most of the sarcomatoid component, compared to strong positivity in squamous nests. The lymph node metastasis in this case displayed an E-cadherin(+) phenotype in the majority of cells with focal low level SNAI1 expression and lack of E-cadherin membrane staining.

**Figure 5:**
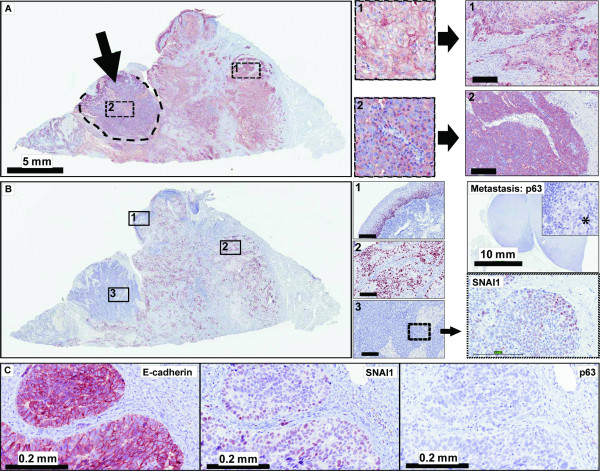
**Immunohistochemical profile of OSCC with sarcomatoid component, Case 2**. **A**, Membranous E-cadherin expression is preserved in the squamous component (1), but lost in the sarcomatoid component (2, arrow). Small images: high power views of the framed areas in the section overview. Corresponding FAK staining (arrow → right) shows accentuation of the invasion front in the squamous component (1), but strong cytoplasmic staining throughout the tumor in the sarcomatoid component (2). Size bars: 0.2 mm. **B**, P63 staining is strong in adjacent mucosa (1) and the squamous tumor component (2), but absent in the sarcomatoid component (3). A magnified area from region 3 shows nuclear SNAI1 positivity (arrow → right). A lymph node metastasis resected at initial presentation is completely negative for p63 (see overview and magnified insert; asterisk indicating the carcinoma). **C**, A temporal fossa metastasis 26 months after initial presentation is positive for E-cadherin and SNAI1, but negative for p63.

The second case was a 63 year old female with a T2N2cM0 poorly differentiated SCC of the tongue. Lymphatic/vascular, but no perineural invasion was reported. 26 months after surgery of the primary tumor this patient developed a right temporal fossa mass lesion. Resected tissue from this lesion confirmed distant recurrence of the SCC. Subsequently a right upper lobe lung mass was detected as well. The patient was referred for palliative care and died after a total follow-up of 28 months. Slide review revealed a distinct, well circumscribed area within the primary tumor displaying E-cadherin(-), p63(-), SNAI1(+), FAK(+) phenotype (Figure 5 and Additional File 5). The lymph node metastasis in this case was strikingly p63 negative, but displayed SNAI1 expression in only 10% of all tumor cells. E-cadherin was cytoplasmic with almost complete absence of membrane staining. AE1/AE3 produced weak to moderate staining and Vimentin was positive in ~90% cells in the sarcomatoid component of the primary tumor. The metastasis from the right temporal fossa was characterized by a strong E-cadherin membrane staining and strong AE1/AE3 reactivity in the bulk of the tissue. However, significant nuclear SNAI1 expression was present, focal areas of E-cadherin loss could be discerned and p63 was absent.

## Discussion

We show here that SNAI1-associated EMT is present in OSCC and potentially contributes to tumor progression in at least a subset of tumors. The latter is exemplified by two cases displaying a sarcomatoid tumor component with high level SNAI1 expression. However, SNAI1 expression in tumor cells was an infrequent event in the majority of cases (usually <<5%) and more commonly noted in scattered individual cells or small groups, particularly at the invasion front and in the vicinity of inflammation. Although the presence of a SNAI1-positive tumor component of >10% area was associated with a poorer prognosis in our cohort, the significance of occasional SNAI1-positive cells for patient outcome remains unclear. SNAI1 expression in the tumor stroma was a frequent observation, raising further questions regarding the origin of these cells [19] and the contribution of the stromal compartment to the malignant phenotype [20,21].

Considering the focal nature of EMT we decided to examine complete tissue sections of primary tumors and metastases. With regard to the staining pattern and frequency of SNAI1 expression our results are comparable to other studies previously performed with non-commercial antibodies [22-25]. Two recent studies which examined SNAI1 in HNSCC by IHC were not able to detect substantial expression of the protein in the epithelial tumor component [26,27]. Interestingly, the study by Zidar *et al*. included a group of spindle cell carcinomas which showed SNAI1-positivity in 19/30 cases [26]. In our randomly collected cohort we identified two cases with sarcomatoid component which, although not distinctly of spindle cell type, showed a clearly circumscribed area of SNAI1-positivity with characteristics of a mesenchymal phenotype. It is tempting to speculate that the lymph node metastasis of case 1 and the intracranial metastasis of case 2 show E-cadherin expression due to the reverse process of EMT: mesenchymal-epithelial transition (MET; also epithelial-mesenchymal reverting transition, EMrT). Current research increasingly focuses on both shifts in tumor cell phenotype since re-establishment of cell-cell connections may be crucial for an efficient colonization of the metastatic target organ [28]. Also, it is becoming increasingly accepted that tumor cells may not necessarily undergo a complete transition, but rather seem to adopt different "quasi-mesenchymal" states which, in addition to the transient nature of EMT and the contribution of other E-cadherin repressors, may explain the lack of correlation between SNAI1 expression and the mesenchymal phenotype, including E-cadherin loss.

As in several previous studies (Additional file 6), SNAI1 expression in our cohort was not significantly associated with E-cadherin loss. This observation appears consistent with the proposed focal and/or transient nature of tumor-associated EMT as well as the contribution of additional EMT mediators. Similarly, high cytoplasmic FAK expression was not exclusively found in SNAI1-positive tumors pointing towards other mechanisms which may confer a migratory phenotype. Also consistent with earlier studies, E-cadherin and FAK were most noticeably altered between normal mucosa and tumor [29-32], although a range of different expression levels and considerable intra-tumoral heterogeneity was seen. A marker whose absence might indicate EMT in cancers arising from squamous epithelia is p63. Although overall less intensely labeled than in basal and parabasal cells of normal mucosa, p63 was generally preserved in OSCC. However, in two poorly differentiated OSCC a complete absence of p63 expression in the sarcomatoid component was found indicating a more complete transition towards the mesenchymal phenotype.

Interest in the role of EMT and its reverse process, MET, in tumor progression has increased exponentially in the last few years [33], although the clinical significance of the phenomenon has been a matter of continuing debate [4]. One obstacle has been the fact that *in vivo *proof of the phenomenon has been difficult to obtain. The reasons for this may be manifold, one possibly being the focal and transient nature of the process, which may employ a number of different mediators besides SNAI1 [34]. Since the initial description of the E-cadherin repressor SNAI1 as potential mediator of a more aggressive tumor phenotype [35], several studies have examined the potential consequences of EMT in SCC of the head and neck (HNSCC) *in vitro *[36-38] and *in vivo *[25,26,32,39,40] using a range of different methods. Some of the confusion over the prevalence of SNAI1 expression in human cancers may also be derived from the use of different antibodies and IHC scoring methods leading to obvious differences particularly with regard to the subcellular compartment considered positive for SNAI1 staining (Additional file 6). Thus, we carefully examined the specificity of the SNAI1 antibodies used in our study employing different controls and analytical methods.

## Conclusions

Reactivation of components of the developmental EMT program likely contributes to tumor progression in at least a subset of OSCC. Although SNAI1 expression is an infrequent event, its presence in a significant proportion of tumor cells may be associated with a poor prognosis and can herald the presence of a sarcomatoid component. P63 may be negative in this situation which can complicate histopathologic diagnosis in cases with occult primary tumor. Further investigation of other E-cadherin repressors (e.g. ZEB1) including a larger cohort will help to elucidate the contribution of EMT to tumor progression in OSCC and other cancers.

## Competing interests

The authors declare that they have no competing interests with regard to this work.

## Authors' contributions

JS conceived the study, participated in data analysis and drafted the manuscript. GB collected the patient cohort, participated in the collection of clinical data and contributed to the manuscript. JCH tested the antibodies and performed the immunohistochemistry staining. BP-O and DWH participated in data analysis. JCI collected clinical data and participated in data analysis. WRG contributed to study design and coordination, and helped to draft the manuscript. All authors read and approved the final manuscript.

## Pre-publication history

The pre-publication history for this paper can be accessed here:

http://www.biomedcentral.com/1472-6890/10/1/prepub

## Supplementary Material

Additional file 1 **Controls and antibody specificity**. **A**, SiHa and ME180 xenografts grown in SCID mice show mesenchymal and squamous characteristics, respectively. Sections of both xenografts were used as positive and negative controls. **B**, Placenta, SiHa xenograft and spindle cell carcinoma (SpCC) of the head and neck were used to test SNAI1 antibody specificity in a competition assay. Absence of staining after incubation with SC10432P blocking peptide confirms specific staining in cell nuclei. **C**, SiRNA treatment of SiHa cells confirms antibody specificity in an immunoblot performed with AF3639 on SiHa monolayer lysates. **D**, Comparison of AF3639 and SC10432 shows identical nuclear staining of the extravillous trophoblast. IHC with SC10432 additionally results in some focal cytoplasmic staining (asterisk) which can also be seen inside the villi in section B of this figure. Click here for file

Additional file 2 **High level SNAI1 expression is associated with poor outcome**. **A**, DSS and **C**, EFS are not significantly shortened by low level SNAI1 (≥ 5%) expression in our cohort. However, SNAI1-positivity in >10% cells of primary tumor and/or metastasis is associated with significantly shorter **B**, DSS and **D**, EFS by univariate analysis (log rank test). Click here for file

Additional file 3 **FAK, E-cadherin and p63 expression are specifically altered in tumors and metastases**. **A**, A significant inverse relationship was found between E-cadherin and FAK_c _expression. Spearman's rank correlation and 95% confidence interval are shown. The relationship was not significant for tumors only. **B**, FAKc; **C**, E-cadherin and **D**, p63 are significantly different in tumors and metastases compared to mucosa adjacent to tumor. An arrow in D indicates the p63-negative metastasis of case 2. **E**, p63 is not significantly altered with respect to SNAI1 expression levels in the majority of OSCC. However, two cases with sarcomatoid component show p63 loss and SNAI1-positivity. (Open and black symbols in diagrams B-E correspond to case 1 and 2, respectively). Click here for file

Additional file 4 **OSCC with sarcomatoid component, Case 1**. **A**, Slide overview and magnified insert illustrate the morphology of the primary tumor in case #1 (haematoxylin & eosin). **B**, Staining with AE1/AE3 cytokeratin cocktail results in strong labeling of the squamous component (arrow), weak-absent labeling of the sarcomatoid component (arrowheads) and absence of labeling in the stroma (asterisk). Click here for file

Additional file 5 **OSCC with sarcomatoid component, Case 2**. **A**, Slide overview and magnified insert illustrates the morphology of the primary tumor in case #2 (haematoxylin & eosin). Arrowhead in insert indicates the sarcomatoid, arrow the squamous component. **B**, Staining with AE1/AE3 cytokeratin cocktail results in strong-moderate labeling of the squamous component (arrow), moderate labeling of the sarcomatoid component (arrowhead) and absence of labeling in the stroma (asterisk). Click here for file

Additional file 6 **Supplemental table**. Studies on SNAI1 performed with immunohistochemistry on FFPE tissue specimens. Click here for file
