# Supplementary figures and images for: SNAI1 expression and the mesenchymal phenotype: an immunohistochemical study performed on 46 cases of oral squamous cell carcinoma
Source: BMC Clin Pathol. 2010 Feb 5;10:1. doi: 10.1186/1472-6890-10-1 (PMC2829523; doi:10.1186/1472-6890-10-1)

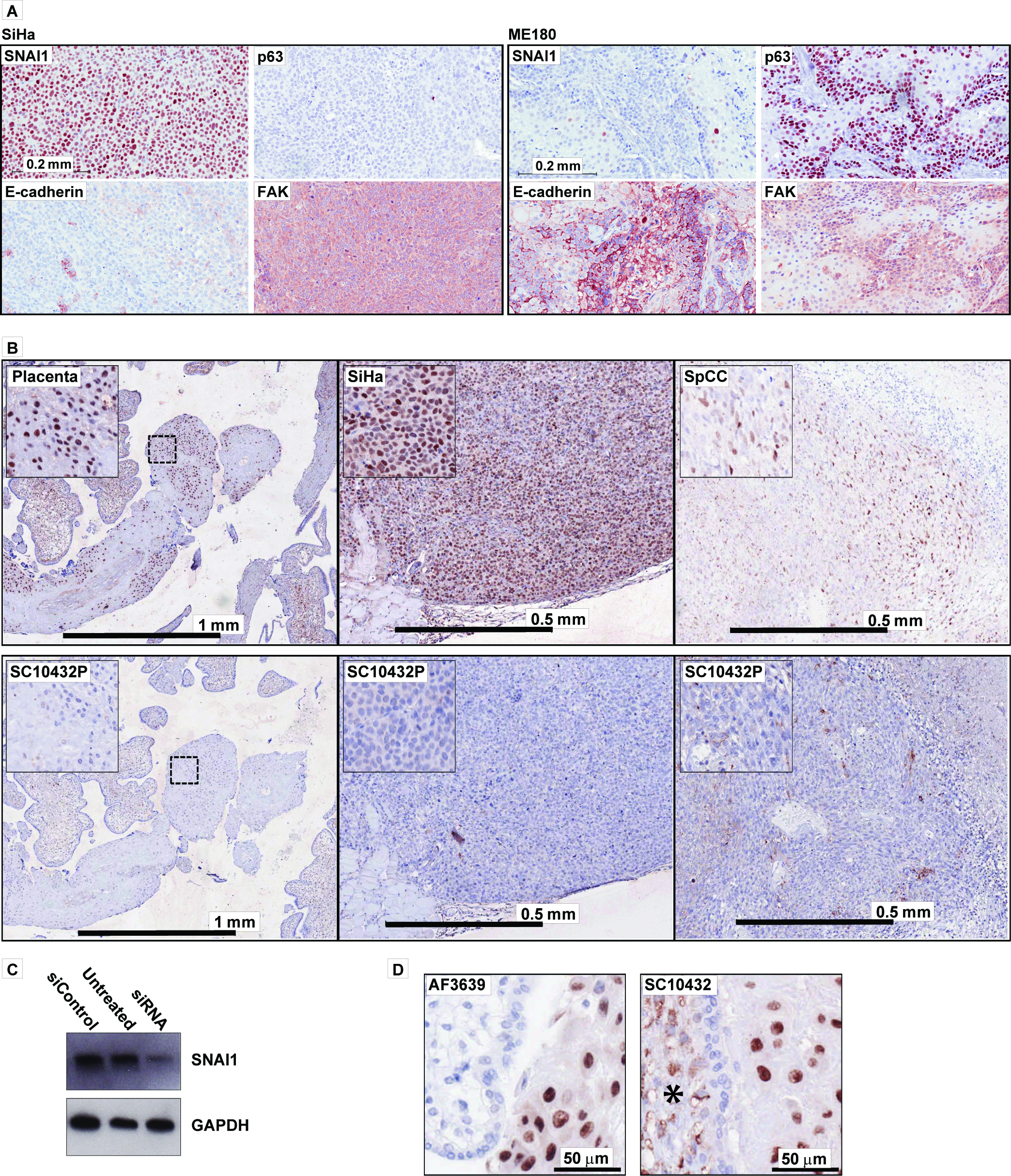

Supplement: Additional file 1 — Controls and antibody specificity. A, SiHa and ME180 xenografts grown in SCID mice show mesenchymal and squamous characteristics, respectively. Sections of both xenografts were used as positive and negative controls. B, Placenta, SiHa xenograft and spindle cell carcinoma (SpCC) of the head and neck were used to test SNAI1 antibody specificity in a competition assay. Absence of staining after incubation with SC10432P blocking peptide confirms specific staining in cell nuclei. C, SiRNA treatment of SiHa cells confirms antibody specificity in an immunoblot performed with AF3639 on SiHa monolayer lysates. D, Comparison of AF3639 and SC10432 shows identical nuclear staining of the extravillous trophoblast. IHC with SC10432 additionally results in some focal cytoplasmic staining (asterisk) which can also be seen inside the villi in section B of this figure. [file 1472-6890-10-1-S1.TIFF]

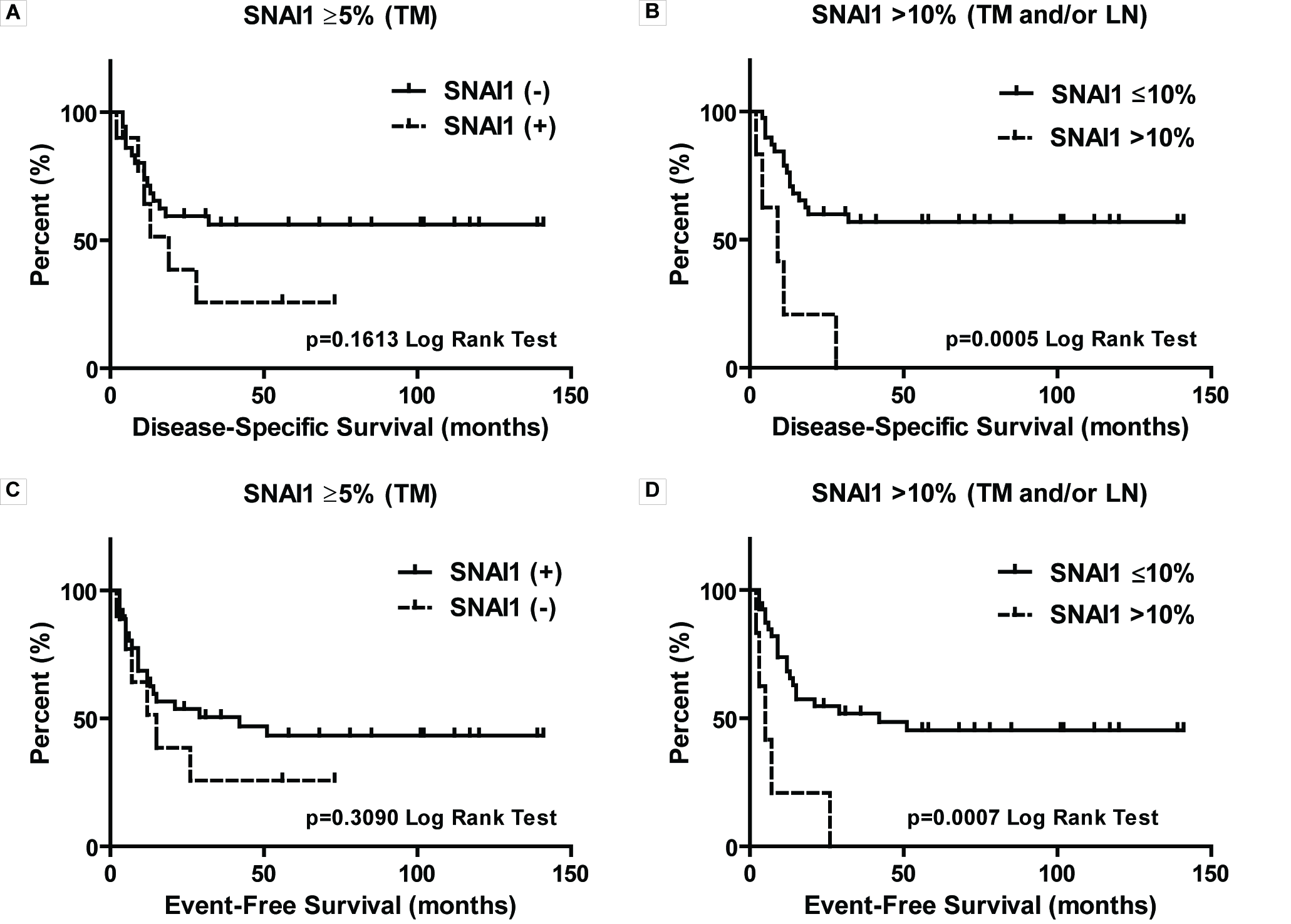

Supplement: Additional file 2 — High level SNAI1 expression is associated with poor outcome. A, DSS and C, EFS are not significantly shortened by low level SNAI1 (≥ 5%) expression in our cohort. However, SNAI1-positivity in >10% cells of primary tumor and/or metastasis is associated with significantly shorter B, DSS and D, EFS by univariate analysis (log rank test). [file 1472-6890-10-1-S2.TIFF]

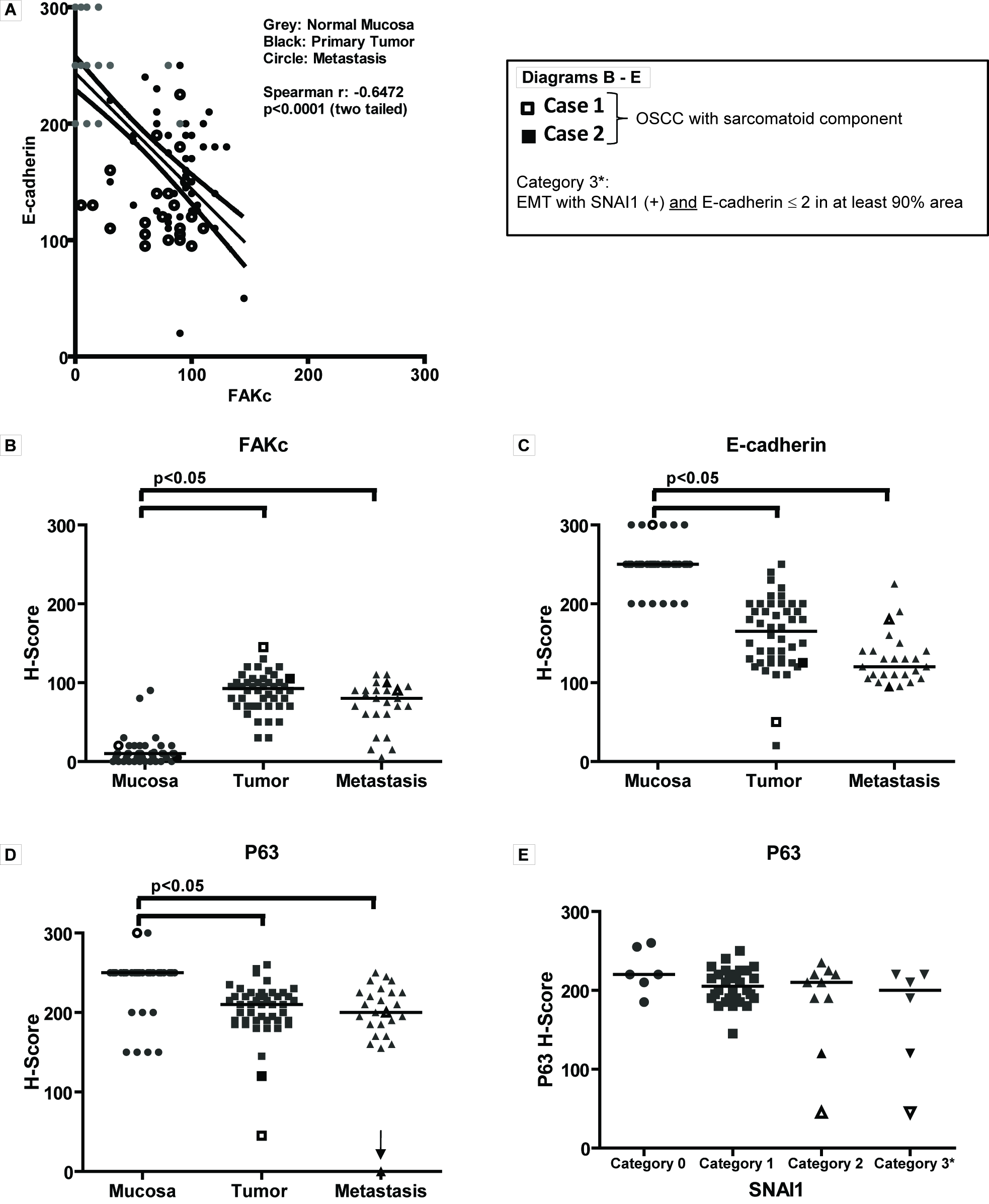

Supplement: Additional file 3 — FAK, E-cadherin and p63 expression are specifically altered in tumors and metastases. A, A significant inverse relationship was found between E-cadherin and FAKc expression. Spearman's rank correlation and 95% confidence interval are shown. The relationship was not significant for tumors only. B, FAKc; C, E-cadherin and D, p63 are significantly different in tumors and metastases compared to mucosa adjacent to tumor. An arrow in D indicates the p63-negative metastasis of case 2. E, p63 is not significantly altered with respect to SNAI1 expression levels in the majority of OSCC. However, two cases with sarcomatoid component show p63 loss and SNAI1-positivity. (Open and black symbols in diagrams B-E correspond to case 1 and 2, respectively). [file 1472-6890-10-1-S3.TIFF]

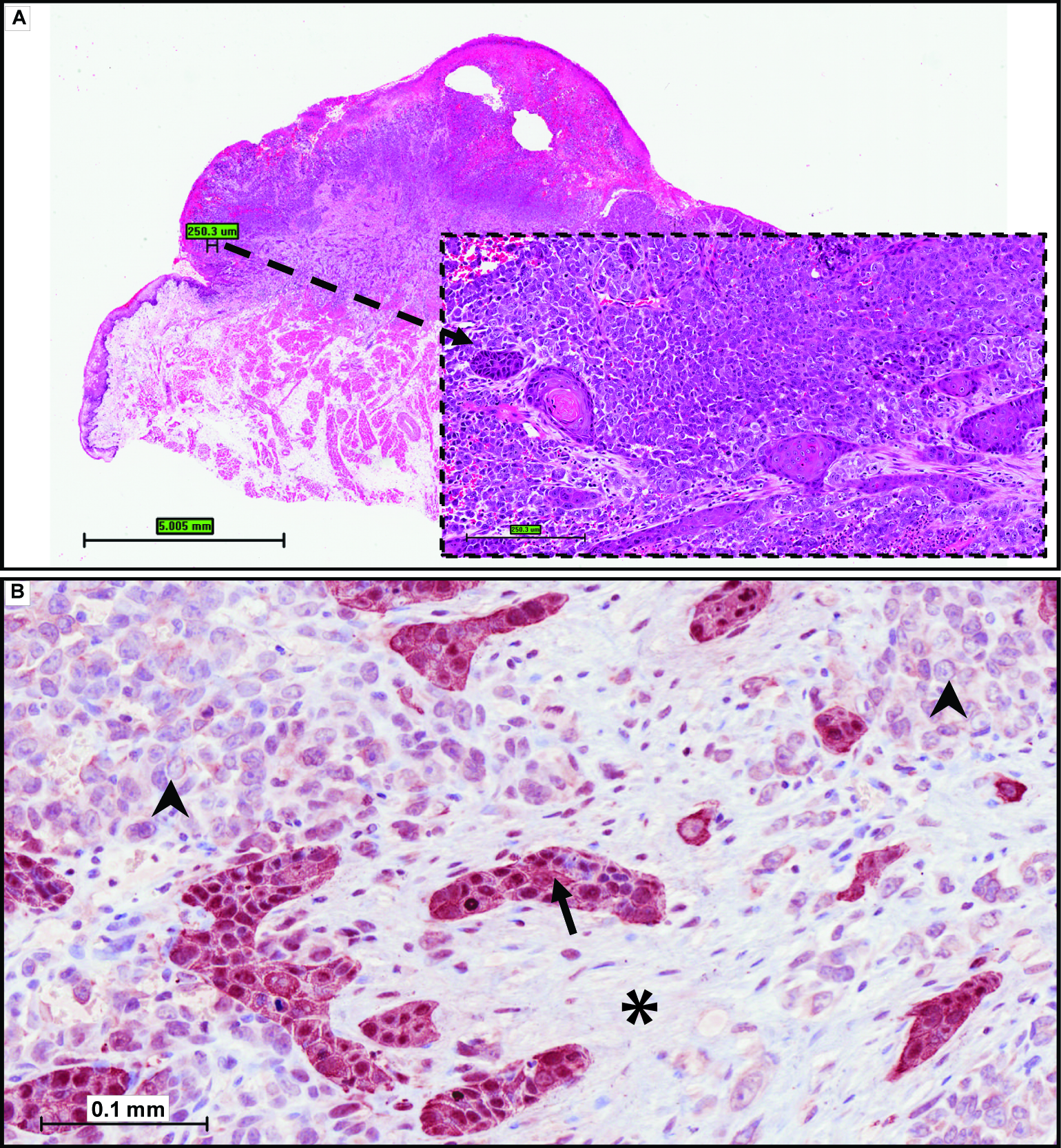

Supplement: Additional file 4 — OSCC with sarcomatoid component, Case 1. A, Slide overview and magnified insert illustrate the morphology of the primary tumor in case #1 (haematoxylin & eosin). B, Staining with AE1/AE3 cytokeratin cocktail results in strong labeling of the squamous component (arrow), weak-absent labeling of the sarcomatoid component (arrowheads) and absence of labeling in the stroma (asterisk). [file 1472-6890-10-1-S4.TIFF]

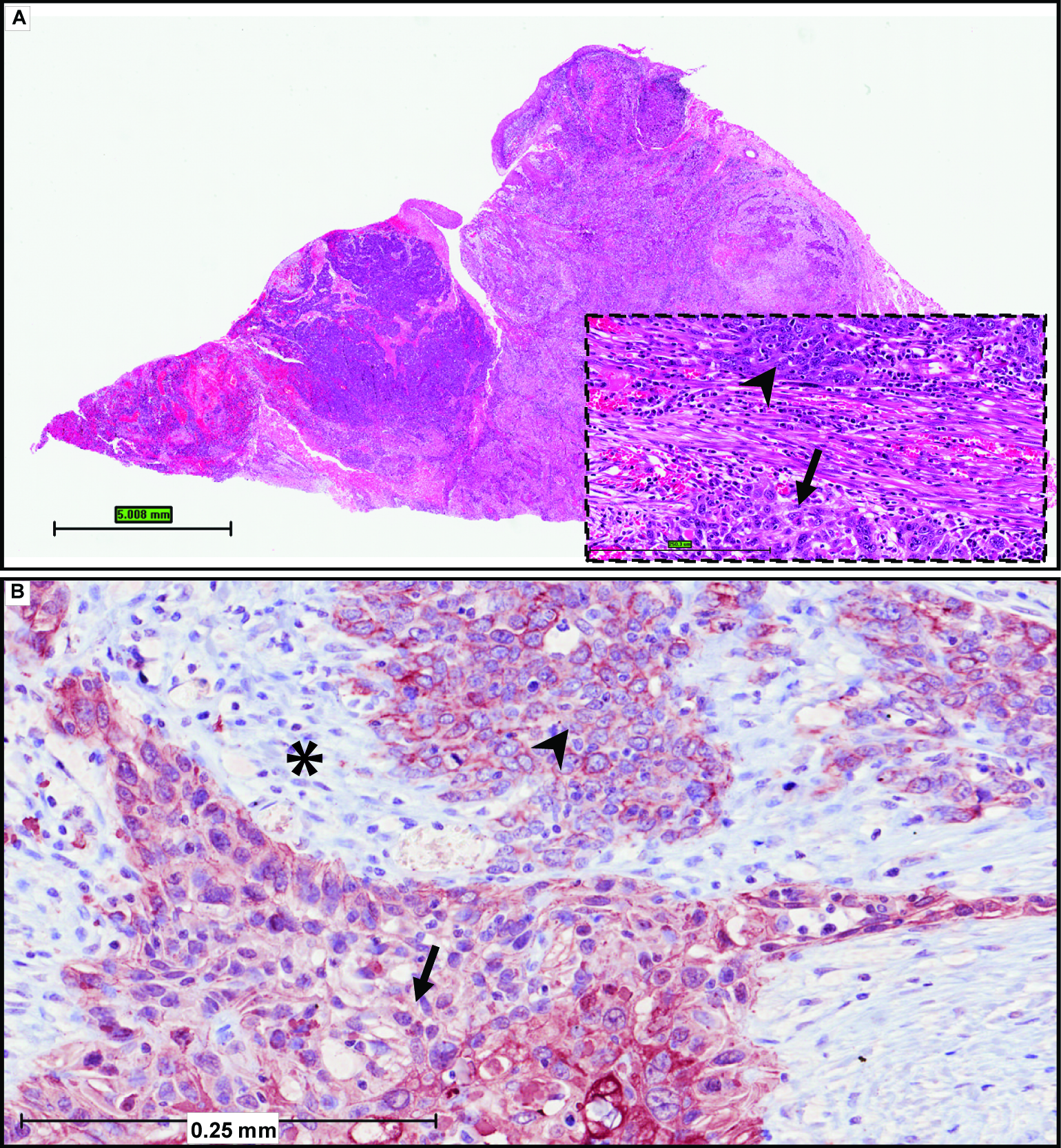

Supplement: Additional file 5 — OSCC with sarcomatoid component, Case 2. A, Slide overview and magnified insert illustrates the morphology of the primary tumor in case #2 (haematoxylin & eosin). Arrowhead in insert indicates the sarcomatoid, arrow the squamous component. B, Staining with AE1/AE3 cytokeratin cocktail results in strong-moderate labeling of the squamous component (arrow), moderate labeling of the sarcomatoid component (arrowhead) and absence of labeling in the stroma (asterisk). [file 1472-6890-10-1-S5.TIFF]
