# Supplementary material for: SNAI1 expression and the mesenchymal phenotype: an immunohistochemical study performed on 46 cases of oral squamous cell carcinoma
Source: BMC Clin Pathol. 2010 Feb 5;10:1. doi: 10.1186/1472-6890-10-1 (PMC2829523; doi:10.1186/1472-6890-10-1)
Supplement: Additional file 6 — Supplemental table. Studies on SNAI1 performed with immunohistochemistry on FFPE tissue specimens. [file 1472-6890-10-1-S6.DOC]

**Table 2: Studies on SNAI1 performed with immunohistochemistry on FFPE tissue specimens**.

| **Study** | **Tumor Type** | **SNAI1 Antibody** | **IHC Staining** | **Study Features** | **Significant Findings** |
| --- | --- | --- | --- | --- | --- |
| Rosivatz E  et al.,  Virchows Arch. 2006 Mar;448 (3):277-87.[1] | Adenocarcinomas of the upper gastrointestinal tract (esophagus, cardia, stomach) | Sn9H2 | **Nuclear** | Tissue Microarray  N=340 | - SNAI1-positive: 7.9% (27/340) - 14/27 SNAI1-positive cases reported with 5-25% immunoreactivity in tumor cells - No correlation between SNAI1 and E-cadherin. - No correlation with clinicopathological parameters. |
| Franci C  et al.,  Oncogene. 2006 Aug 24;25(37):5134-44.[2] | Cervical squamous cell carcinoma, adenocarcinoma of the colon, fibrosarcoma, sarcoma, infantile fibromatosis | MAb EC3 | **Nuclear** | Tumor biopsies | - High SNAI1 expression in fibrosarcomas and sarcomas. - SNAI1 expression in epithelial tumors restricted to stromal cells in vicinity of the tumor and tumoral cells in the same area. |
| Blechschmidt K  et al.,  Diagn Mol Pathol. 2007 Dec;16(4):222-8.[3] | Endometrioid adenocarcinoma of the endometrium | Sn9H2 | **Nuclear** | N=87 primary tumors and 26 unrelated metastases | - SNAI1-positive: 28.7% of primary tumors and 53.8% of metastases - Positive if >5% immunoreactive - SNAI1 in metastases correlated with higher grade and reduced E-cadherin. |
| Blechschmidt K  et al.,  Br J Cancer. 2008 Jan 29;98(2):489-95.[4] | Ovarian cancer | Sn9H2 | **Nuclear** | N=48 primary tumors and 50 metastases | - SNAI1-positive: 37.5% of primary tumors and 52% of metastases - Positive if ≥1% immunoreactive - Borderline significant difference in overall survival with SNAI1 expression in metastases. - No correlation between SNAI1 and E-cadherin. |
| Yang MH  et al.,  Nat Cell Biol. 2008 Mar;10  (3):295-305.[5] | Head and Neck squamous cell carcinoma | sc-82199,  Santa Cruz Biotech. | **Nuclear** | Tissue Microarray  N=147 primary tumors and 56 metastases | - SNAI1-positive: 37.4% of primary tumors and 82.1% of metastatic tumors - Positive if >50% immunoreactive - SNAI1 correlated with shorter metastasis-free period and reduced overall survival. |
| Usami Y  et al.,  J Pathol. 2008 Jul;215(3):330-9.[6] | Esophageal squamous cell carcinoma | Abcam | **Nuclear** | N=72 | - SNAI1-positive: 38% (27/72) - Positive if ≥20% immunoreactive - Elevated SNAI1 expression at the invasive front associated with lymphatic and venous vessel invasion, lymph node metastases, and tumor stage. |
| Zidar N  et al.,  Virchows Arch. 2008 Sep;453 (3):267-74. [7] | Head and Neck squamous cell carcinoma | Sn9H2 | **Nuclear** | N=30 Spindle cell carcinomas (SpCC)  N=30 Moderately differentiated squamous cell carcinomas (SCC) | - SNAI1-positive: 19/30 SpCC - SNAI1-positive: 4/30 SCC (occasional tumor cells) - Positive if immunoreactive (no lower limit reported) - No correlation between SNAI1 and E-cadherin. |
| Franci C  et al.,  PLoS One. 2009;4(5):e5595. Epub 2009 May 18.[8] | Colorectal cancer | MAb EC3 | **Nuclear** | Tissue Microarray  N=162 | - SNAI1-positive: 79% (128/162) - Positive if ≥1% immunoreactive - SNAI1 expression in stroma correlated with specific survival. |
| Jin H  et al.,  Int J Cancer. 2009 Sep 30. [Epub ahead of print][9] | Ovarian cancer | Abcam | **Nuclear** (late stage tumors) & **Nuclear-cytoplasmic** (early stage tumors) | Tissue Microarray  N=41 serous adenocarcinomas with 14 matched metastatic tumors, 12 serous borderline tumors, 5 cystadenomas, 4 normal controls | - Range of SNAI1 immunoreactivity lowest in normal/benign and highest in tumor samples. |
| Peinado H  et al.,  Cancer Res. 2008 Jun 15;68(12):4541-50.[10] | Laryngeal squamous cell carcinoma | MAb EC3 | **Nuclear and Cytoplasmic** | Tissue Microarray  N=256 | - SNAI1-positive: 16% (40/251) with 3% (8/251) high-positive - Positive if ≥5% immunoreactive, High-positive if >15% immunoreactive - Correlation between SNAI1 and LOXL2 expression. - No association between SNAI1 and disease-free/overall survival. |
| Zhou BP  et al.,  Nat Cell Biol. 2004 Oct;6(10):931-40.[11] | Breast cancer | Santa Cruz Biotech. | Compartment not reported (Figure shown with predominantly **cytoplasmic** staining) | N=129 | - SNAI1-positive: 56% (72/129); 17 cases low expression and 55 cases high expression - Positive if immunoreactive (no lower limit reported) - SNAI1 correlated with inhibition of GSK-3β and E-cadherin downregulation. - SNAI1 correlated with metastasis. |
| Roy HK  et al.,  Dig Dis Sci. 2005 Jan;50(1):42-6.[12] | Colorectal cancer | T-18 and E-18,  Santa Cruz Biotech. | Compartment not reported (Figure shown with predominantly **cytoplasmic** staining) | Tissue Microarray  N=59 | - SNAI1-positive: 78% (46/59) - Positive if immunoreactive (no lower limit reported) - Trend towards increased presence of SNAI1 in tumors with distant metastases. |
| Natsugoe S  et al.,  Oncol Rep. 2007 Mar;17(3):517-23.[13] | Esophageal squamous cell carcinoma | E-18, Santa Cruz Biotech. | **Cytoplasmic and Perinuclear** | N=194 | - SNAI1-positive: 61.7% (84/194) - Positive if >10% immunoreactive - SNAI1 associated with deep invasion, increased lymph node metastases, and advanced stage. - No correlation between SNAI1 and E-cadherin. |
| Yang MH  et al.,  Oncogene. 2007 Mar 1;26(10):1459-67.[14] | Head and Neck squamous cell carcinoma | sc-82199,  Santa Cruz Biotech. | **Cytoplasmic** | Tissue Microarray  N=147 primary tumors and 56 metastatic tumor samples | - SNAI1-positive: 37.4% primary tumors - Positive if ≥25% immunoreactive - SNAI1 expression associated with lymph node metastasis. - NBS1/SNAI1 co-expression indicated short metastasis-free period and overall survival. |
| Kim MA  et al.,  Histopathology. 2009 Mar;54(4):442-51.[15] | Gastric carcinoma | Santa Cruz Biotech. | **Cytoplasmic** | Tissue Microarray  N=598 | - SNAI1-positive: 42.9% (245/571) - Positive if >10% immunoreactive - SNAI1 associated with invasion and lymph node metastasis. - SNAI1 was independent indicator of prognosis by multivariate analysis. |
| Waldmann J  et al.,  Ann Surg Oncol. 2009 Jul;16 (7):1997-2005.[16] | Pheochromocytoma | Santa Cruz Biotech. | **Cytoplasmic** | N=44 primary tumors, 3 lymph node metastases and 2 peritoneal metastases | - SNAI1-positive: 28% (13/47) - Positive if ≥5% immunoreactive; strong positive if >50% immunoreactive - SNAI1 associated with malignancy. |
| Bruyere F  et al.,  Urol Oncol. 2009 Jan 20. [Epub ahead of print][17] | Transitional cell carcinoma of the bladder | sc-28199,  (H-130),  Santa Cruz Biotech. | Compartment not reported (Figure shown with predominantly **cytoplasmic** staining) | Tissue Microarray  N=87 | - Strong SNAI1-positive: 43.7%; Weak SNAI1-positve: 56.3% - Positive if ≥1% immunoreactive - SNAI1 prognostic for tumor recurrence by uni- and multivariate analysis. |
| Waldmann J  et al.,  Br J Cancer. 2008 Dec 2;99(11):1900-7.[18] | Adrenocortical carcinoma | Santa Cruz Biotech. | Compartment not assessable | N=26 primary tumors, two lymph node metastases and one liver metastasis | - SNAI1-positive: 65% (17/26) primary tumors; Strong positive: 7 tumors at the invasion front and 2/3 metastases - Positive if ≥5% immunoreactive; strong positive if >50% immunoreactive - SNAI1 associated with advanced stage, decreased survival rates and higher risk for distant metastases. |

References

1. Rosivatz E, Becker KF, Kremmer E, Schott C, Blechschmidt K, Hofler H, Sarbia M: **Expression and nuclear localization of Snail, an E-cadherin repressor, in adenocarcinomas of the upper gastrointestinal tract**. *Virchows Arch* 2006, **448**(3):277-287.

2. Franci C, Takkunen M, Dave N, Alameda F, Gomez S, Rodriguez R, Escriva M, Montserrat-Sentis B, Baro T, Garrido M *et al*: **Expression of Snail protein in tumor-stroma interface**. *Oncogene* 2006, **25**(37):5134-5144.

3. Blechschmidt K, Kremmer E, Hollweck R, Mylonas I, Hofler H, Kremer M, Becker KF: **The E-cadherin repressor snail plays a role in tumor progression of endometrioid adenocarcinomas**. *Diagn Mol Pathol* 2007, **16**(4):222-228.

4. Blechschmidt K, Sassen S, Schmalfeldt B, Schuster T, Hofler H, Becker KF: **The E-cadherin repressor Snail is associated with lower overall survival of ovarian cancer patients**. *Br J Cancer* 2008, **98**(2):489-495.

5. Yang MH, Wu MZ, Chiou SH, Chen PM, Chang SY, Liu CJ, Teng SC, Wu KJ: **Direct regulation of TWIST by HIF-1alpha promotes metastasis**. *Nat Cell Biol* 2008, **10**(3):295-305.

6. Usami Y, Satake S, Nakayama F, Matsumoto M, Ohnuma K, Komori T, Semba S, Ito A, Yokozaki H: **Snail-associated epithelial-mesenchymal transition promotes oesophageal squamous cell carcinoma motility and progression**. *J Pathol* 2008, **215**(3):330-339.

7. Zidar N, Gale N, Kojc N, Volavsek M, Cardesa A, Alos L, Hofler H, Blechschmidt K, Becker KF: **Cadherin-catenin complex and transcription factor Snail-1 in spindle cell carcinoma of the head and neck**. *Virchows Arch* 2008, **453**(3):267-274.

8. Franci C, Gallen M, Alameda F, Baro T, Iglesias M, Virtanen I, Garcia de Herreros A: **Snail1 protein in the stroma as a new putative prognosis marker for colon tumours**. *PLoS ONE* 2009, **4**(5):e5595.

9. Jin H, Yu Y, Zhang T, Zhou X, Zhou J, Jia L, Wu Y, Zhou BP, Feng Y: **Snail is critical for tumor growth and metastasis of ovarian carcinoma**. *Int J Cancer* 2009.

10. Peinado H, Moreno-Bueno G, Hardisson D, Perez-Gomez E, Santos V, Mendiola M, de Diego JI, Nistal M, Quintanilla M, Portillo F *et al*: **Lysyl oxidase-like 2 as a new poor prognosis marker of squamous cell carcinomas**. *Cancer Res* 2008, **68**(12):4541-4550.

11. Zhou BP, Deng J, Xia W, Xu J, Li YM, Gunduz M, Hung MC: **Dual regulation of Snail by GSK-3beta-mediated phosphorylation in control of epithelial-mesenchymal transition**. *Nat Cell Biol* 2004, **6**(10):931-940.

12. Roy HK, Smyrk TC, Koetsier J, Victor TA, Wali RK: **The transcriptional repressor SNAIL is overexpressed in human colon cancer**. *Dig Dis Sci* 2005, **50**(1):42-46.

13. Natsugoe S, Uchikado Y, Okumura H, Matsumoto M, Setoyama T, Tamotsu K, Kita Y, Sakamoto A, Owaki T, Ishigami S *et al*: **Snail plays a key role in E-cadherin-preserved esophageal squamous cell carcinoma**. *Oncol Rep* 2007, **17**(3):517-523.

14. Yang MH, Chang SY, Chiou SH, Liu CJ, Chi CW, Chen PM, Teng SC, Wu KJ: **Overexpression of NBS1 induces epithelial-mesenchymal transition and co-expression of NBS1 and Snail predicts metastasis of head and neck cancer**. *Oncogene* 2007, **26**(10):1459-1467.

15. Kim MA, Lee HS, Lee HE, Kim JH, Yang HK, Kim WH: **Prognostic importance of epithelial-mesenchymal transition-related protein expression in gastric carcinoma**. *Histopathology* 2009, **54**(4):442-451.

16. Waldmann J, Slater EP, Langer P, Buchholz M, Ramaswamy A, Walz MK, Schmid KW, Feldmann G, Bartsch DK, Fendrich V: **Expression of the transcription factor snail and its target gene twist are associated with malignancy in pheochromocytomas**. *Ann Surg Oncol* 2009, **16**(7):1997-2005.

17. Bruyere F, Namdarian B, Corcoran NM, Pedersen J, Ockrim J, Voelzke BB, Mete U, Costello AJ, Hovens CM: **Snail expression is an independent predictor of tumor recurrence in superficial bladder cancers**. *Urol Oncol* 2009.

18. Waldmann J, Feldmann G, Slater EP, Langer P, Buchholz M, Ramaswamy A, Saeger W, Rothmund M, Fendrich V: **Expression of the zinc-finger transcription factor Snail in adrenocortical carcinoma is associated with decreased survival**. *Br J Cancer* 2008, **99**(11):1900-1907.
